# Antioxidant and neurodevelopmental gene polymorphisms in prematurely born individuals influence hypoxia-related oxidative stress

**DOI:** 10.1038/s41598-024-65647-4

**Published:** 2024-06-28

**Authors:** Katja Goričar, Tadej Debevec, Vita Dolžan, Agnès Martin, Vincent Pialoux, Grégoire P. Millet, Damjan Osredkar

**Affiliations:** 1 Pharmacogenetics Laboratory, Institute of Biochemistry and Molecular Genetics, Faculty of Medicine, University of Ljubljana, Ljubljana, Slovenia; 2 Faculty of Sport, University of Ljubljana, Ljubljana, Slovenia; 3 Department of Automatics, Biocybernetics and Robotics, Jožef Stefan Institute, Jamova Cesta 39, 1000 Ljubljana, Slovenia; 4 Univ Lyon, Laboratoire Interuniversitaire de Biologie de la Motricité EA 7424, Université Claude Bernard Lyon 1, Faculté de Médecine Rockefeller, 69008 Lyon, France; 5 Institute of Sport Sciences, University of Lausanne, Lausanne, Switzerland; 6 Department of Pediatric Neurology, University Children’s Hospital Ljubljana, University Medical Centre Ljubljana, Bohoričeva 20, 1525 Ljubljana, Slovenia; 7 Center for Developmental Neuroscience, Medical Faculty, University of Ljubljana, Ljubljana, Slovenia

**Keywords:** Physiology, Biochemistry, Genetic association study

## Abstract

Preterm born (PTB) infants are at risk for injuries related to oxidative stress. We investigated the association between antioxidant and neurodevelopmental gene polymorphisms and oxidative stress parameters in PTB male young adults and their term-born counterparts at rest and during exercise. Healthy young PTB (N = 22) and full-term (N = 15) males underwent graded exercise tests in normobaric normoxic (F_i_O_2_ = 0.21) and hypoxic (F_i_O_2_ = 0.13) conditions. *CAT* rs1001179 was associated with decrease in nitrites in the whole group and in PTB individuals (P = 0.017 and P = 0.043, respectively). *GPX1* rs1050450 was associated with decrease in ferric reducing antioxidant power in the whole group and in full-term individuals (P = 0.017 and P = 0.021, respectively). *HIF1A* rs11549465 was associated with decrease in nitrotyrosine and increase in malondialdehyde (P = 0.022 and P = 0.018, respectively). *NOTCH4* rs367398 was associated with increase in advanced oxidation protein products and nitrites (P = 0.002 and P = 0.004, respectively) in hypoxia. In normoxia, *NOTCH4* rs367398 was associated with increase in malondialdehyde in the whole group (P = 0.043). *BDNF* rs6265 was associated with decreased nitrites/nitrates in the whole group and in PTB individuals (P = 0.009 and P = 0.043, respectively). Polymorphisms in investigated genes and PTB might influence oxidative stress response after exercise in normoxic or hypoxic conditions far beyond the neonatal period in young male adults.

## Introduction

Preterm birth (PTB) related complications are the leading cause of death in children under 5 years of age^1^. In the lower-income countries, 12% of babies are born prematurely compared to 9% in higher-income countries^2^. Due to tremendous advances in perinatal medicine leading to improved survival of newborns born prematurely^3^, the impact of long-term consequences of PTB remains an important health issue^4^. The pathophysiological mechanisms involved in PTB consequences are multiple, among which oxidative stress has an important role^5,6^.

Oxidative cellular processes are inherently associated to the production of free radicals, including reactive oxygen (ROS) and nitrogen species (RNS)^7^, which play an important role in cell signalling^8^. However, ROS and RNS overproduction and/or insufficient antioxidant defence can disrupt the redox balance, leading to oxidative stress characterised by cellular damage by oxidation and nitration^9^. Multiple factors modulate oxidative stress level, including chronic psychological stress^10^, circadian rhythm dysregulation^11^, physical exercise^6,12^, nutrition^13–15^, hypoxia^16^, infection^17^, environmental toxins^18^, and others. While preterm newborns are highly susceptible to oxidative stress^7^, only a limited number of clinical studies have investigated the relationship between oxidative stress, which commonly accompanies the clinical constellation of preterm birth, and long-term consequences in adolescents and adults born prematurely^6,19^. While ROS have been previously studied in prematurely born adult individuals^6^, showing their higher resistance to oxidative stress response to exercise in hypoxia, the underlying genetic polymorphisms in selected antioxidant and neurodevelopmental genes has not yet been studied.

Compared to term born, preterm infants are more at risk for injuries related to oxidative and nitrosative stress due exposure to high oxygen concentrations, inflammation, high levels of free iron, and immaturity of antioxidant systems^7^. Most of the complications of prematurity, such as bronchopulmonary dysplasia (BPD), retinopathy of prematurity (ROP), necrotizing enterocolitis (NEC), intraventricular hemorrhage (IVH), periventricular leukomalacia (PVL), and punctate white matter lesions (PWML), appear related to oxidative stress^20^. Whether this increased vulnerability of PTB translates to increased vulnerability for oxidative stress in adult life is not clear and the data are inconclusive. Innate genetic mechanisms have a role in regulating response to oxidative stress^21,22^, but the clinical implications of these are still largely unknown. Nevertheless, evidence has consistently shown that adult survivors of PTB have increased risks of chronic disorders involving various organ systems, including cardiovascular, endocrine/metabolic, respiratory, renal, neurodevelopmental, and psychiatric disorders, which either persist from childhood into adulthood or sometimes first manifest in adulthood^23^.

The aim of the present study was to investigate the oxidative stress responses to acute exercise in normobaric normoxia and in hypoxia in individuals born pre-term as compared to their age and aerobic capacity matched counterparts full-term born. We hypothesized that common functional genetic polymorphisms in selected antioxidant (superoxide dismutase 2 (*SOD2)*, catalase (*CAT)*, glutathione peroxidase 1 (*GPX1)* and hypoxia-inducible factor 1-alpha (*HIF1A*)) and neurodevelopmental (neurogenic locus notch homolog 4 (*NOTCH4*) and brain-derived neurotrophic factor (*BDNF*)) genes are associated the relative change in oxidative stress markers in response to acute exercise. This could then elucidate whether genetic factors play a role in oxidative stress management in pre-term born individuals during normoxia and/or hypoxia.

## Methods

### Participants

The participants’ characteristics and detailed explanation of the study protocol have been outlined previously^6,24^. Briefly, thirty-seven healthy young males provided written informed consent and participated in this study. Twenty-two participants were born pre-term (inclusion criteria: gestational age ≤ 32 weeks and gestational body mass ≤ 1500 g) and fifteen were born full-term. All participants were near sea level residents, apparently healthy and free of any chronic cardiorespiratory and/or haematological diseases. They were not exposed to altitudes ≥ 1500 m for at least one-month preceding the study.

### Exercise testing

Two graded exercise tests on an electromagnetically braked cycle-ergometer (Ergo Bike Premium, Daum electronics, Fürth, Germany) were performed in a randomized and blinded manner by all participants on two separate occasions under the following conditions: (1) Normobaric normoxic (F_i_O_2_ = 0.21; P_i_O_2_ = 147 mmHg) and (2) normobaric hypoxic (F_i_O_2_ = 0.13; P_i_O_2_ = 91 mmHg) conditions. The tests were always performed at the same time of the day. Following a resting period, and the initial warmup period at 60W the workload was increased for 40 W/min^−1^ until volitional exhaustion.

The methodological details of the cardiorespiratory measurements during both normoxic and hypoxic incremental exercise tests are detailed elsewhere^24^. To obtain gas exchange and ventilatory variables, the participants breathed through a facemask (Vmask, 7500 series, Hans Rudolph Inc., Shawnee, USA) attached to a metabolic cart (Quark CPET, Cosmed, Rome, Italy) throughout the test duration. Peripheral oxygen saturation (SpO_2_) was measured transcutaneously using a finger pulse oximetry device (Nellcor, BCI 3301, Boulder, USA).

### Blood sampling and biochemical analysis

For genetic analyses, peripheral blood samples were collected on a separate morning visit to the lab with participants fasted overnight. Five ml sample was obtained from seated participants into a tube with sodium citrate and immediately stored at − 80 °C.

As detailed previously^25^, additional blood sampling (6 mL of venous blood) was performed immediately before and within 1 min following the cessation of each incremental exercise test. Blood samples were drawn into EDTA collection tubes, immediately centrifuged (10 min at 3500 rpm, 4 °C) with the plasma aliquoted into 1.5 mL cryotubes and subsequently immediately frozen to − 20 °C and to − 80 °C.

Genomic DNA was isolated from peripheral blood leukocytes using the FlexiGene DNA kit (Qiagen, Hilden, Germany) according to the manufacturer’s instructions. *CAT* rs1001179, *SOD2* rs4880, *HIF1A* rs11549465, *HIF1A* rs11549467, *NOTCH4* rs367398, and *BDNF* rs6265 single nucleotide polymorphisms (SNPs) were genotyped using competitive allele specific PCR (KASP) assays (KBiosciences, Hoddesdon, UK and LGC Genomics, Hoddesdon, UK), while *GPX1* rs1050450 was genotyped using TaqMan genotyping assay (Thermo Fisher Scientific, Waltham, MA, USA) according to the manufacturer’s instructions.

The exact details of the biochemical analysis for determination of Oxidative stress markers: advanced oxidation protein products (AOPP), malondialdehyde (MDA), ferric reducing antioxidant power (FRAP), nitrates and nitrites as well as activities of antioxidant enzymes CAT, GPX1 and SOD2 have been detailed previously^6^. All spectrophotometry and fluometry measurements were performed with TECAN Infinite 2000 plate reader (Männedorf, Switzerland).

### Statistical analysis

Categorical and continuous variables were described using frequencies and median with 25–75% range, respectively. Relative change was defined as the difference in oxidative stress markers after exercise test and before exercise test, divided by the value before exercise test. For all investigated polymorphisms, we determined the minor allele frequency (MAF) and assessed the deviation from Hardy–Weinberg equilibrium (HWE) using the standard chi-square test. Dominant genetic model was used in all analyses. In the subgroup analysis, polymorphisms with ≤ 3 participants in one of the categories were excluded from the analyses. For the associations with antioxidant enzyme activity, only SNPs in the corresponding genes (*CAT*, *GPX1* and *SOD2*) were included in the analysis. Non-parametric Mann–Whitney test was used to evaluate the association of investigated polymorphisms with relative change of oxidative stress parameters.

As six SNPs were included in the analyses, Bonferroni correction was used to account for multiple comparisons: P-values below 0.008 were considered statistically significant, while P-values between 0.008 and 0.050 were considered nominally significant. All statistical tests were two-sided. The statistical analysis was performed using IBM SPSS Statistics version 27.0 (IBM Corporation, Armonk, NY, USA).

### Ethical approval and consent to participate

The overall study protocol was pre-registered at ClinicalTrials.gov (NCT02780908), approved by the National Committee for Medical Ethics at the Ministry of Health of the Republic of Slovenia (0120-101/2016-2) and conducted in line with the guidelines of the Declaration of Helsinki.

## Results

For participants born full-term, mean age was 22 (± 2) years, height 180 (± 5) cm, weight 73 (± 6) kg, mean BMI was 22 (± 1) kg/m^2^, and mean gestational age was 39 (± 2) weeks. For participants born pre-term, mean age was 21 (± 1) years, height 175 (± 8) cm, weight 69 (± 8) kg, mean BMI was 22 (± 3) kg/m^2^, and mean gestational age was 29 (± 3) weeks. Except for weight and gestational age at birth, the two groups did not differ significantly^24^.

The relative change of oxidative stress parameters after exercise test performed in normoxic or hypoxic conditions is presented in Supplementary Table S1. A larger decrease of nitrotyrosine levels was observed in full-term participants compared to pre-term participants in normoxic conditions (P = 0.022), while the relative change of other parameters did not differ between both groups.

Genotype frequencies of selected polymorphisms in antioxidant and neurodevelopmental genes are presented in Table 1. All SNPs were in agreement with HWE. Due to the low MAF, *HIF1A* rs11549467 was not included in any further analyses.

**Table 1:** Genotype frequencies of selected SNPs in antioxidant and neurodevelopmental genes.

SNP	Function	Genotype	N (%)	MAF	pHWE
Antioxidant genes					
* CAT* rs1001179	c.-262C>T	CC	22 (59.5)	0.22	0.479
		CT	14 (37.8)		
		TT	1 (2.7)		
* GPX1* rs1050450	p.Pro200Leu	CC	20 (54.1)	0.28	0.409
		CT	13 (35.1)		
		TT	4 (10.8)		
* SOD2* rs4880	p.Val16Ala	CC	9 (24.3)	0.55*	0.275
		CT	15 (40.5)		
		TT	13 (35.1)		
* HIF1A* rs11549465	p.Pro582Ser	CC	31 (83.8)	0.09	0.199
		CT	5 (13.5)		
		TT	1 (2.7)		
* HIF1A* rs11549467	p.Ala588Thr	GG	36 (97.3)	0.01	0.934
		GA	1 (2.7)		
		AA	0 (0.0)		
Neurodevelopmental genes					
* NOTCH4* rs367398	c.-25G>A	GG	11 (29.7)	0.41	0.156
		GA	22 (59.5)		
		AA	4 (10.8)		
* BDNF* rs6265	p.Val66Met	GG	18 (48.6)	0.30	0.832
		GA	16 (43.2)		
		AA	3 (8.1)		

*Variant allele frequency.

MAF: minor allele frequency, HWE: Hardy–Weinberg equilibrium, SNP: single nucleotide polymorphism.

### Antioxidant genes

*CAT*, *GPX1* and *SOD2* polymorphisms were not associated with change of enzyme activities for catalase, glutathione peroxidase and superoxide dismutase, respectively (Supplementary Table S2). In the analysis performed in all participants, a nominally significant decrease in nitrites was observed in carriers of at least one polymorphic *CAT* rs1001179 allele in exercise test performed in hypoxic conditions, compared to an increase in carriers of two polymorphic alleles (P = 0.017, Supplementary Table S2). Additionally, a larger decrease in FRAP was observed in carriers of at least one polymorphic *GPX1* rs1050450 allele in hypoxic conditions compared to carriers of two polymorphic alleles (P = 0.017). In exercise test performed in hypoxic conditions, a nominally significant decrease in nitrotyrosine and increase in MDA was observed in carriers of at least one polymorphic *HIF1A* rs11549465 allele (P = 0.022 and P = 0.018, respectively; Table 2). No significant differences were observed for polymorphisms in antioxidant genes in normoxic conditions.

**Table 2:** Association of selected SNPs in antioxidant and neurodevelopmental genes with relative change (%) of oxidative stress parameters after graded exercise test (all participants, N = 37).

Condition	Parameter	*HIF1A* rs11549465			*NOTCH4* rs367398			*BDNF* rs6265		
		CC	CT + TT	P	GG	GA + AA	P	GG	GA + AA	P
Normoxia	FRAP	− 9.6 (− 13.8 to − 2.4)	− 6.1 (− 37.7 to 1.4)	1.000	− 5.9 (− 34 to − 2.4)	− 9.6 (− 13.7 to − 1)	0.707	− 10 (− 13.9 to − 3.8)	− 5.1 (− 16.3 to 0.6)	0.499
	AOPP	32.7 (0.6–73.6)	70.2 (4.4–113.7)	0.302	32.7 (4.7–99.1)	41.1 (− 0.1 to 76.9)	0.756	44.2 (− 2.4 to 68.1)	32.7 (5.7–88.7)	0.578
	MDA	1 (− 11.9 to 31.4)	− 10 (− 24 to 42.1)	0.733	− 1.2 (− 31.9 to − 0.7)	12.2 (− 10.1 to 46.3)	0.043	− 1.1 (− 13.7 to 43.8)	11.7 (− 21.4 to 31.4)	0.730
	Nitrotyrosine	− 17.2 (− 56.3 to 12.3)	− 24.9 (− 77.1 to − 7.4)	0.302	− 19.8 (− 62.9 to 12.3)	− 17.3 (− 51.1 to 12.2)	0.731	− 12 (− 37.6 to 19.6)	− 27.4 (− 58.9 to 4)	0.258
	Nitrites	15.1 (− 5.2 to 43.2)	33.8 (− 22 to 189.6)	0.442	15.7 (− 7.4 to 47.4)	16 (− 11.5 to 48.6)	1.000	16.1 (− 11.5 to 67.4)	15.7 (− 7.4 to 37.4)	0.552
	Nitrites/nitrates	15.7 (− 8.4 to 34.2)	29.3 (− 4.9 to 59.6)	0.363	25.9 (− 5 to 39.5)	14.2 (− 9.6 to 37)	0.481	− 3.6 (− 14 to 26.3)	26.5 (0.3–73.4)	0.009
Hypoxia	FRAP	− 5.2 (− 20.7 to 9.3)	− 14.3 (− 47.8 to 0.3)	0.231	− 9.4 (− 42.4 to 4.9)	− 5 (− 20.7 to 9.9)	0.544	− 3.3 (− 20.7 to 12)	− 7.3 (− 24.5 to − 0.1)	0.408
	AOPP	23.3 (7.7–34.8)	25.2 (8.5–63.9)	0.644	7.7 (− 17.7 to 17.7)	25.4 (16.4–45.9)	0.002	21.7 (12.7–45.9)	23.7 (5.1–31.9)	0.443
	MDA	− 1.2 (− 13.7 to 29.9)	39.4 (17.3–83.5)	0.022	2.6 (− 14.2 to 23.4)	8.7 (− 10.5 to 36.4)	0.589	12.4 (− 10.6 to 30.4)	1.8 (− 13.7 to 42.5)	0.893
	Nitrotyrosine	2.5 (− 55.8 to 28)	− 73.9 (− 81.2 to − 46.6)	0.018	− 55.2 (− 73.6 to 29.8)	0.2 (− 72.4 to 21.5)	0.710	− 12.1 (− 62 to 24)	− 32.1 (− 74.8 to 26.2)	0.628
	Nitrites	− 3.2 (− 16.6 to 26.3)	− 2.6 (− 15.4 to 129.9)	0.820	− 26.9 (− 38.8 to − 3.5)	7.3 (− 8 to 28.4)	0.004	6.8 (− 7.9 to 33.1)	− 7.4 (− 28.2 to 10.2)	0.055
	Nitrites/nitrates	11.8 (− 5.8 to 42.4)	12.5 (− 1.9 to 103.3)	0.605	12 (− 17.2 to 41.7)	11.6 (− 1.9 to 59.2)	0.612	29.6 (− 0.4 to 81.4)	8.1 (− 8.5 to 23)	0.055

AOPP: advanced oxidation protein products, FRAP: ferric reducing antioxidant power, MDA: malondialdehyde, SNP: single nucleotide polymorphism.

**Figure 1:**
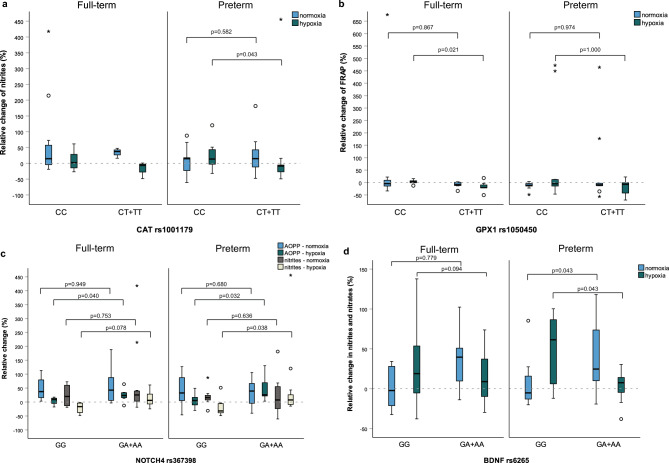
Association with the relative change (%) of selected oxidative stress parameters after graded exercise test for different genotypes of *CAT* rs1001179 (**A**), *GPX1* rs1050450 (**B**), *NOTCH4* rs367398 (**C**), and *BDNF* rs6265 (**D**). P-values are not reported for polymorphisms with ≤ 3 participants in one of the categories in subgroups. Circles represent outliers and stars (*) represent extreme outliers.

We also evaluated the association of selected polymorphisms with the relative change of oxidative stress parameters after exercise test separately in participants born full-term (Table 3) or pre-term (Table 4). A decrease in nitrites was observed in carriers of at least one polymorphic *CAT* rs1001179 allele in hypoxic conditions in pre-term participants (P = 0.043, Fig. 1A). For *GPX1* rs1050450, a larger decrease in FRAP in carriers of at least one polymorphic allele in hypoxic conditions was seen only in participants born full-term (P = 0.021), while no differences were observed in pre-term participants (P = 0.262, Fig. 1B). Additionally, *GPX1* rs1050450 and *SOD2* rs4880 were nominally associated with relative change in nitrotyrosine levels in hypoxic condition in participants born full-term (P = 0.029 and P = 0.012, respectively, Table 3).

**Table 3:** Association of selected SNPs in antioxidant genes with relative change (%) of oxidative stress parameters after graded exercise test in full-term participants (N = 15).

Condition	Parameter	*GPX1* rs1050450			*SOD2* rs4880		
		CC	CT + TT	P	CC	CT + TT	P
Normoxia	Enzyme activity (GPX1 or SOD2)*	− 19.2 (− 38.3 to 0.2)	− 10.2 (− 17.4 to − 0.8)	0.281	9.3 (− 16.5 to 33.9)	41.3 (7.2–89.4)	0.145
	FRAP	− 4.4 (− 17.8 to 21.8)	− 7.9 (− 12.9 to 0.5)	0.867	− 12.3 (− 34 to 1)	− 4.4 (− 10.6 to 11.2)	0.181
	AOPP	54.5 (3.3–88.7)	33.6 (4.3–132.5)	0.779	25.8 (1.2–56.5)	54.5 (13.9–137.3)	0.272
	MDA	11.7 (− 21.4 to 22.2)	24.1 (− 0.9 to 64.9)	0.189	− 0.8 (− 14.2 to 23.5)	22.2 (− 3.8 to 64.7)	0.272
	Nitrotyrosine	− 48.9 (− 62.9 to − 19.8)	− 30.9 (− 75 to 6)	0.536	− 28.9 (− 40.1 to 33.8)	− 58 (− 81.7 to − 18.2)	0.145
	Nitrites	37.4 (− 7.4 to 47.4)	20.7 (2.6–62.1)	1.000	27.9 (13.1–46.2)	2.2 (− 12.9 to 130.9)	0.607
	Nitrites/nitrates	34.2 (− 2.1 to 56.1)	− 8.3 (− 25 to 28.8)	0.054	6 (− 29.6 to 47.9)	26.5 (− 4.9 to 42.5)	0.328
Hypoxia	Enzyme activity (GPX1 or SOD2)*	− 17.4 (− 31.3 to − 9.8)	− 34.9 (− 41.3 to − 16.7)	0.336	− 12.3 (− 22.1 to 50.3)	− 11.5 (− 32.8 to 20.5)	0.864
	FRAP	2.4 (− 1.3 to 9.3)	− 20.7 (− 21 to − 6.6)	0.021	− 12 (− 20.8 to 3.6)	− 0.1 (− 18.7 to 7.1)	0.689
	AOPP	17 (8.3–29.3)	19.4 (13.6–34.8)	0.397	23.5 (6.8–34.7)	17 (8.9–27.8)	0.607
	MDA	1.8 (− 16.7 to 34.3)	21.7 (− 8.7 to 45.7)	0.536	37.6 (4.5–276.7)	1.8 (− 30 to 28.3)	0.088
	Nitrotyrosine	− 76.8 (− 94.5 to − 57.3)	− 4.5 (− 68.8 to 34.9)	0.029	18.2 (− 37.5 to 168.9)	− 74.1 (− 92.5 to − 55)	0.012
	Nitrites	− 6 (− 21.6 to 0.9)	13.6 (− 20.2 to 29.5)	0.281	− 2.8 (− 25.3 to 11.8)	− 1.9 (− 14.5 to 28.6)	0.607
	Nitrites/nitrates	10.9 (0–41.7)	14.1 (− 25.4 to 63.4)	0.613	20.6 (− 8.3 to 73.9)	10.9 (− 15.9 to 37.2)	0.776

*The activity of the enzyme, encoded by the corresponding gene: e.g. glutathione peroxidase activity for *GPX1* rs1050450 and superoxide dismutase activity for *SOD2* rs4880.

AOPP: advanced oxidation protein products, FRAP: ferric reducing antioxidant power, MDA: malondialdehyde, SNP: single nucleotide polymorphism.

**Table 4:** Association of selected SNPs in antioxidant genes with relative change (%) of oxidative stress parameters after graded exercise test in pre-term participants (N = 22).

Condition	Parameter	*CAT* rs1001179			*GPX1* rs1050450		
		CC	CT + TT	P	CC	CT + TT	P
Normoxia	Enzyme activity (CAT or GPX1)*	91.3 (− 1.2 to 149.7)	68.9 (19.7–87.2)	0.872	− 15.5 (− 39.1 to − 5)	− 23.5 (− 28.4 to − 10.5)	0.896
	FRAP	− 12.1 (− 14.8 to 42.5)	− 6.9 (− 30.3 to − 3.5)	0.974	− 11.2 (− 15.3 to − 3.2)	− 9.6 (− 24.4 to 86.9)	0.744
	AOPP	32.4 (− 9.4 to 100.8)	45.4 (5–66.7)	0.674	23 (− 12 to 71.3)	64.1 (18.7–84.4)	0.357
	MDA	5.7 (− 11.6 to 29.6)	− 1.2 (− 47.4 to 39.5)	0.582	− 0.7 (− 45.9 to 25.4)	− 1.5 (− 13.7 to 52.7)	0.695
	Nitrotyrosine	8.1 (− 19.7 to 32.2)	− 12.6 (− 62.1 to 11.1)	0.140	− 6.5 (− 30.6 to 17.8)	1.9 (− 36.9 to 17.7)	0.845
	Nitrites	14.5 (− 34.2 to 42)	14.8 (− 17.7 to 49.1)	0.582	14 (− 23.3 to 48.3)	16.7 (− 36.9 to 48.1)	0.972
	Nitrites/nitrates	14.2 (− 8 to 23.9)	23.5 (− 7.5 to 73.7)	0.702	20.6 (− 4.8 to 32.7)	0.3 (− 16.1 to 79.3)	0.431
Hypoxia	Enzyme activity (CAT or GPX1)*	12 (− 11.1 to 49.4)	45.2 (0.1–102.2)	0.314	− 15.3 (− 32.5 to 3.4)	− 25.6 (− 33.1 to − 17)	0.556
	FRAP	− 6 (− 22.9 to 8.7)	− 8.3 (− 23.2 to 18.1)	1.000	− 5.2 (− 14.5 to 12.2)	− 7.3 (− 45.6 to 3.2)	0.262
	AOPP	22.5 (− 4 to 46.3)	24.6 (5.9–67.1)	0.539	23.8 (5.1–37.6)	39.7 (6.4–86.6)	0.471
	MDA	− 5.5 (− 12.3 to 16.5)	14 (− 10.6 to 36.3)	0.254	− 1.2 (− 11.6 to 18.8)	25.4 (− 14.9 to 56.6)	0.512
	Nitrotyrosine	14.2 (− 6.7 to 33)	− 54.7 (− 73.6 to 27.4)	0.173	16.3 (− 43.2 to 35.5)	− 27.2 (− 75 to 22.6)	0.193
	Nitrites	13.4 (− 4.8 to 45.4)	− 8.6 (− 38.6 to − 3.5)	0.043	− 5.6 (− 26.4 to 7.6)	16 (− 12.8 to 85.8)	0.247
	Nitrites/nitrates	8.9 (− 5.8 to 34.5)	20.5 (5.3–79.6)	0.426	9.5 (− 6.2 to 27.8)	20.5 (1.2–89.2)	0.169

*The activity of the enzyme, encoded by the corresponding gene: e.g. catalase activity for *CAT* rs1001179 and glutathione peroxidase activity for *GPX1* rs1050450.

AOPP: advanced oxidation protein products, FRAP: ferric reducing antioxidant power, MDA: malondialdehyde, SNP: single nucleotide polymorphism.

### Neurodevelopmental genes

In the analysis performed in all participants, *NOTCH4* rs367398 was significantly associated with change in AOPP and nitrites in exercise test performed in hypoxic conditions (P = 0.002 and P = 0.004, respectively, Table 2). A higher increase in AOPP was observed in carriers of at least one polymorphic *NOTCH4* rs367398 allele, while nitrites tended to increase in carriers of at least one polymorphic allele and decrease in carriers of two normal alleles. Additionally, MDA levels increased only in carriers of at least one polymorphic *NOTCH4* rs367398 allele in normoxic conditions, but the difference was only nominally significant (P = 0.043). On the other hand, nitrites and nitrates tended to increase in carriers of at least one polymorphic *BDNF* rs6265 allele and decrease in carriers of two normal alleles in exercise test performed in normoxic conditions (P = 0.009, Table 2).

The association of selected polymorphisms with the relative change of oxidative stress parameters separately in participants born full-term or pre-term is presented in Tables 5 and 6, respectively. A higher increase in AOPP was observed in carriers of at least one polymorphic *NOTCH4* rs367398 allele in hypoxic conditions in both full-term and pre-term participants (P = 0.040 and P = 0.032, respectively, Fig. 1C). In hypoxic conditions, nitrites tended to increase in carriers of at least one polymorphic *NOTCH4* rs367398 allele and decrease in carriers of two normal alleles in both full-term and pre-term participants, but the difference was nominally significant only in pre-term participants (P = 0.078 and P = 0.038, respectively, Fig. 1C). For *BDNF* rs6265, an increase in nitrites and nitrates in normoxic conditions in carriers of at least one polymorphic allele was nominally significant only in participants born pre-term (P = 0.043), while no differences were observed in full-term participants (P = 0.094, Fig. 1D). On the other hand, a larger increase in nitrites and nitrates was observed for carriers of two normal *BDNF* rs6265 alleles in hypoxic conditions in pre-term participants (P = 0.043, Fig. 1D).

**Table 5:** Association of selected SNPs in neurodevelopmental genes with relative change (%) of oxidative stress parameters after graded exercise test in in full-term participants (N = 15).

Condition	Parameter	*NOTCH4* rs367398			*BDNF* rs6265		
		GG	GA + AA	P	GG	GA + AA	P
Normoxia	FRAP	− 14.3 (− 30 to − 5.9)	− 4.4 (− 11.2 to 2.9)	0.177	− 7.9 (− 12.9 to − 0.9)	− 4.4 (− 17.8 to 2.9)	0.779
	AOPP	37.2 (9.6–96.4)	42.7 (2.8–88.7)	0.949	33.6 (2.9–52.3)	86.9 (9–113.3)	0.336
	MDA	− 10.2 (− 27.2 to 16.4)	16.8 (− 0.4 to 57.4)	0.104	5.7 (− 9.2 to 64.9)	16.8 (− 21.4 to 31.5)	0.779
	Nitrotyrosine	− 48.4 (− 78.7 to − 23.4)	− 30 (− 58.9 to − 12.3)	0.343	− 30.9 (− 72.5 to 4.1)	− 58 (− 62.9 to − 16.5)	0.536
	Nitrites	20 (− 16 to 66.4)	25.4 (2.2–41.8)	0.753	10.1 (− 13.3 to 38.9)	37.4 (− 1 to 214.4)	0.281
	Nitrites/nitrates	26.2 (4.9–36.3)	− 2.7 (− 14 to 45.4)	0.661	− 2.4 (− 25 to 28.8)	39.5 (− 7.2 to 56.1)	0.094
Hypoxia	FRAP	2.4 (− 9.5 to 14.8)	− 16.4 (− 20.9 to 2.4)	0.138	− 18.5 (− 20.8 to 4.3)	− 1.3 (− 12.6 to 9.3)	0.536
	AOPP	8.9 (− 11.2 to 15.7)	23.7 (14.5–34.7)	0.040	15.8 (10.5–30.4)	23.7 (8.3–31.9)	0.694
	MDA	− 3 (− 36.7 to 19.3)	32.8 (− 7.2 to 46.8)	0.226	8.5 (− 12.1 to 30.2)	34.3 (− 16.7 to 46.8)	0.613
	Nitrotyrosine	− 75.9 (− 97.3 to 15.4)	− 52.8 (− 76.8 to 15.5)	0.412	− 38.6 (− 83.3 to 19.5)	− 74.1 (− 90.6 to 3.9)	0.463
	Nitrites	− 16.5 (− 43.1 to − 2.9)	6 (− 7.4 to 29.2)	0.078	0 (− 7.2 to 29.5)	− 1.9 (− 24.7 to 21.3)	0.397
	Nitrites/nitrates	58.1 (− 4.2 to 121.9)	8.8 (− 12.3 to 32.5)	0.138	18.8 (− 8.8 to 64)	8.8 (− 19.5 to 41.7)	0.779

AOPP: advanced oxidation protein products, FRAP: ferric reducing antioxidant power, MDA: malondialdehyde, SNP: single nucleotide polymorphism.

**Table 6:** Association of selected SNPs in neurodevelopmental genes with relative change (%) of oxidative stress parameters after graded exercise test in pre-term participants (N = 22).

Condition	Parameter	*NOTCH4* rs367398			*BDNF* rs6265		
		GG	GA + AA	P	GG	GA + AA	P
Normoxia	FRAP	− 5.1 (− 48.6 to 3.8)	− 11.2 (− 14.3 to − 4.1)	0.447	− 11.6 (− 16.3 to − 6.4)	− 5.5 (− 15.4 to − 1.9)	0.456
	AOPP	32.7 (4.7–99.1)	39.5 (− 6.8 to 66.8)	0.680	61.1 (− 9.4 to 79)	29.1 (5–73.9)	0.872
	MDA	− 1.2 (− 44.4 to − 0.7)	1 (− 17.8 to 44.3)	0.332	− 4.2 (− 47.8 to 20.5)	0.2 (− 28.3 to 30.8)	0.418
	Nitrotyrosine	7.5 (− 56.3 to 17.1)	− 6.5 (− 30 to 18.5)	0.837	7.1 (− 20.9 to 32.2)	− 12.5 (− 49.8 to 11.1)	0.254
	Nitrites	15.7 (0.7–30.3)	7.6 (− 29.3 to 58.1)	0.636	26.4 (− 13.6 to 78)	7.4 (− 29.4 to 17.3)	0.169
	Nitrites/nitrates	20.6 (− 14.1 to 93.2)	15.7 (− 5.9 to 27.4)	0.783	− 5.2 (− 13.3 to 18.6)	24.6 (5.1–73.7)	0.043
Hypoxia	FRAP	− 14.8 (− 46.8 to − 5.2)	− 1.4 (− 14.2 to 12.7)	0.066	3.2 (− 9.8 to 15.1)	− 11.8 (− 37.9 to − 2.9)	0.080
	AOPP	5.2 (− 25.5 to 25.4)	26 (20.1–73)	0.032	32.3 (17.8–81.7)	15.8 (5–43.5)	0.283
	MDA	2.6 (− 8.9 to 37.7)	− 1.4 (− 13.7 to 29.9)	0.581	18.8 (− 12.3 to 30.4)	− 1.3 (− 12.5 to 31)	0.821
	Nitrotyrosine	− 8.9 (− 55.3 to 29.8)	5.8 (− 55.8 to 30)	0.856	9 (− 55.8 to 33)	1.2 (− 55.3 to 27.4)	0.863
	Nitrites	− 32.1 (− 38.8 to − 3.5)	7.5 (− 8.8 to 30.3)	0.038	11.8 (− 10.7 to 62.6)	− 7.7 (− 38.6 to 7.3)	0.114
	Nitrites/nitrates	7.4 (− 17.2 to 16.2)	20.2 (3.1–81.4)	0.079	61.4 (3–87.9)	7.4 (− 5.6 to 16.2)	0.043

AOPP: advanced oxidation protein products, FRAP: ferric reducing antioxidant power, MDA: malondialdehyde, SNP: single nucleotide polymorphism.

## Discussion

The aim of this study was to investigate the association between antioxidant and neurodevelopmental gene polymorphisms and oxidative stress parameters in preterm born male subjects compared to their term born counterparts during normoxic and hypoxic exercise testing. The study examined various oxidative stress parameters, including nitrotyrosine, nitrites, FRAP, MDA, and AOPP. Polymorphisms in antioxidant genes and *HIF1A* were associated with relative change of different oxidative stress parameters only in hypoxic conditions, while polymorphisms in neurodevelopmental genes were associated with change of oxidative stress parameters in both hypoxic and normoxic conditions. Some associations were observed only in pre-term or full-term subjects. As discussed below, the findings of this study suggest that gene polymorphisms in specified genes and preterm birth might influence oxidative stress response after exercise in normoxic/hypoxic conditions far beyond the neonatal period in young adult male subjects.

The study of Filippone et al. found evidence of oxidative stress in the airways of preterm-born adolescents, suggesting long-term respiratory abnormalities after PTB which may be associated with an ongoing airway disease^19^. Our previous work suggested blunted microvascular responsiveness, larger increases in oxidative stress and skeletal muscle oxidative capacity, which may compromise altitude acclimatization in healthy adults born preterm^26^. Higher oxidative stress in adults born preterm could partly be explained by higher consumption of oxygen for every given molecule of ATP production^27^. However, other studies suggest prematurely born adults deal with oxidative stress better than term born counterparts. We have previously shown that in response to exercise in hypoxia, prematurely born adult individuals, compared to term born, exhibit higher resistance to oxidative stress response^6^.

Early-life adversity can also cause epigenetic modifications to the genome that may persist throughout the lifespan. An example of this is the *BDNF* gene, whose methylation has been linked to psychiatric disorders^28^. DNA methylation may also play an important role in long-term consequences of PTB^29^. Furthermore, genetic polymorphisms of antioxidant enzymes can be associated with oxidative stress-associated complications in preterm infants^30^. Hypoxia also significantly affects gene expression as it can trigger transcription and splicing to induce expression of gene sets required for hypoxic adaptation, such as hypoxia-inducible factor (HIF), nuclear factor kappa-light-chain-enhancer of activated B cells (NF-κB), and cAMP response element-binding protein (CREB)^31^. Therefore, interplay of PTB, genetic polymorphisms, and hypoxia induced gene expression/modifications results in modified response to oxidative stress in adults born preterm. As both acute and chronic exercise training increase oxidative stress levels (predominantly within the skeletal muscle and the blood) in a dose-dependent manner^32^, exercise may be used as a way to amplify the response of the innate antioxidant system.

In the present study, a larger decrease of nitrotyrosine levels was observed in full-term born participants compared to pre-term born under normoxic conditions, while the relative change of other oxidative stress parameters did not differ between both groups neither in normoxic nor in hypoxic conditions. Nitrotyrosine, a marker of oxidative stress, is a modified form of the amino acid tyrosine, which is formed by nitration in reaction of tyrosine with reactive nitrogen species such as peroxynitrite^33^. Previous studies reported that nitrotyrosine is increased in preterm born children that developed BPD^34^, however, even though nitrotyrosine levels were increased in cord blood compared to maternal blood, no differences were observed among preterm and full term newborns^35^. Further studies are therefore needed to evaluate the role of tyrosine nitrosylation in response to exercise test.

It has previously been shown that genetic polymorphisms contribute to the development of complications of PTB, such as BPD and IVH. For example, eNOS (*NOS3*) rs2070744 and rs1799983 polymorphisms were independent predictors of an increased risk of developing BPD^36^, while *SOD2* rs8192287 polymorphism was an independent predictor of a decreased risk of developing IVH^30^. However, the association of genetic variability with oxidative stress markers during exercise test in not well known.

Our study investigated several antioxidant genes, including *CAT*, *GPX1*, *SOD2,* and *HIF1A*. Significant associations were found between certain gene polymorphisms and oxidative stress parameters. Carriers of at least one polymorphic *CAT* rs1001179 allele showed a decrease in nitrites and carriers of at least one polymorphic *GPX1* rs1050450 allele exhibited a decrease in FRAP during hypoxic exercise testing. Hypoxia leads to a decreased supply of oxygen, resulting in impaired oxidative phosphorylation in mitochondria, and consequently electron leakage from the electron transport chain increases, leading to enhanced production of ROS^37^. CAT and GPX are key antioxidant enzymes involved in scavenging ROS. Functional genetic variants could influence their antioxidant role: *CAT* rs1001179 is a promotor polymorphism associated with altered gene expression^38^, while *GPX1* rs1050450 is a non-synonymous polymorphism associated with decreased enzyme activity^39^. While their exact roles in hypoxia are still being investigated, these enzymes are likely to play an important role in protecting cells from hypoxia-induced oxidative stress, with the mentioned polymorphisms potentially being protective under hypoxic conditions.

The most studied mechanism of response to hypoxia involves hypoxia inducible factors (HIFs), which are stabilized by low oxygen availability and control the expression of a multitude of genes involved in many cell precesses^40^, including protects against oxidative stress^41^. In our study, carriers of at least one polymorphic *HIF1A* rs11549465 allele showed a significant decrease in nitrotyrosine levels and an increase in MDA during hypoxic exercise testing, but no difference was observed under normoxic conditions. *HIF1A* rs11549465 is a non-synonymous polymorphism that could affect the transcriptional activity of HIF1A and expression of its target genes^42^. As HIF1A is activated during hypoxia, it is not surprising to find the polymorphic allele(s) to play an important role under hypoxic conditions. It has been previously found that hypoxic supramaximal compared to hypoxic low intensity exercise was associated with lover levels of nitrotyrosine in mice^43^, however in this later study this effect was not specifically associated with HIF1A.

We also examined the role of neurodevelopmental gene polymorphisms, specifically promotor *NOTCH4* rs367398 polymorphism and non-synonymous *BDNF* rs6265 that can affect BDNF expression and localization, on oxidative stress parameters. Carriers of at least one polymorphic *NOTCH4* rs367398 allele displayed a significantly higher increase in AOPP, nitrites and MDA during hypoxic exercise testing. Carriers of at least one polymorphic *NOTCH4* rs367398 allele also showed an increase in MDA in normoxic conditions. Notch pathway, including its cross-talk with HIF signaling, is likely to also play an essential role in hypoxia tolerance^44^. In hypoxia-reoxygenation model utilising neonatal rat myocardial cells, Notch1-Nrf2 signaling crosstalk significantly increased cardiomyocyte viability by reduction of the formation of reactive oxygen species and increase of antioxidant activities^45^. How the *NOTCH4* gene polymorphism may contribute to increased oxidative stress in response to hypoxia is not yet clear and warrants further studies. Furthermore, participants carrying at least one polymorphic *BDNF* rs6265 allele showed an increase in nitrites and nitrates during normoxic exercise testing. An interplay between BDNF and oxidative stress has been shown to have an important role in executive function in schizophrenia^46^, while this relationship needs to be further elucidated in hypoxic exercise.

As very little is known about the role of gene polymorphisms of antioxidant and neurodevelopmental genes in PTB, we further analysed our results separately for full-term and preterm born participants to assess any differential effects of gene polymorphisms on oxidative stress parameters after exercise.

Among preterm born participants, carriers of at least one polymorphic *CAT* rs1001179 allele exhibited a decrease in nitrites during hypoxic exercise testing. In full-term born participants, a larger decrease in FRAP was observed in carriers of at least one polymorphic *GPX1* rs1050450 allele during hypoxic conditions, compared to normoxic conditions, while these was not observed in preterm-born participants. These findings suggest potential differences in the influence of gene polymorphisms on antioxidant defence mechanisms between full-term and preterm infants during hypoxia; however these findings need to be tested in larger cohorts.

Both full-term and preterm participants carrying at least one polymorphic *NOTCH4* rs367398 allele showed a higher increase in AOPP during hypoxic exercise testing. In preterm born (but not term born) participants, carriers of at least one polymorphic *BDNF* rs6265 allele displayed a significant increase in nitrites and nitrates during normoxic exercise testing. These results indicate a potential influence of *NOTCH4* and *BDNF* gene polymorphisms on oxidative stress levels in both full-term and preterm infants, with variations in the direction of effect.

It is important to acknowledge some limitations of the study, such as the relatively small sample size and a male only population. In addition, the inclusion of only male participants also limits the generalizability of the obtained results. Thus, causal relationships should not be assumed by the provided data and studies on larger cohorts as well as further investigation into the functional implications of the identified gene polymorphisms are undoubtedly warranted.

## Conclusion

The study provides insights into the associations between antioxidant and neurodevelopmental gene polymorphisms and oxidative stress parameters in preterm and term born male subjects during normoxic and hypoxic exercise testing. The observed differences in oxidative stress levels and gene polymorphism effects highlight the potential role of genetic factors in modulating antioxidant defence mechanisms in response to exercise-induced oxidative stress. Future studies should consider expanding the sample size and exploring functional implications to enhance our understanding of the genetic influences on oxidative stress in preterm infants.

### Supplementary Information

Supplementary Tables.

## Data Availability

The datasets used and/or analyzed during the current study are available from the corresponding author on reasonable request.
