# Supplementary material for: Antioxidant and neurodevelopmental gene polymorphisms in prematurely born individuals influence hypoxia-related oxidative stress
Source: Sci Rep. 2024 Jun 28;14:14956. doi: 10.1038/s41598-024-65647-4 (PMC11213937; doi:10.1038/s41598-024-65647-4)
Supplement: Supplementary file 1 — Supplementary Tables. [file 41598_2024_65647_MOESM1_ESM.docx]

Supplementary Table S1: Relative change (%) of oxidative stress parameters in participants born full-term or pre-term in exercise test performed in normoxic or hypoxic conditions.

| Condition | Parameter | All participants  N=37 | Full-term  N=15 | Pre-term  N=22 | P |
| --- | --- | --- | --- | --- | --- |
| Normoxia | Catalase activity | 56.6 (10.6-117.9) | 44.6 (-5.4-101.3) | 68.9 (12.8-129.2) | 0.417 |
|  | GPX1 activity | -15.5 (-29.4 to -5.2) | -12.5 (-21.1-0.2) | -20.3 (-36.1 to -7) | 0.304 |
|  | SOD2 activity | 12.3 (-8.7-47.1) | 15.5 (3.2-74.7) | 7.6 (-11.2-29.6) | 0.202 |
|  | FRAP | -9.6 (-14.1 to -1.9) | -5.5 (-13.5-0.6) | -9.6 (-14.8 to -3.1) | 0.435 |
|  | AOPP | 39.5 (3-81.6) | 42.7 (3.3-88.7) | 36.1 (-3.2-74.3) | 0.511 |
|  | MDA | -0.4 (-18.5-31.5) | 11.8 (-11.9-43.7) | -1 (-35-29.6) | 0.213 |
|  | Nitrotyrosine | -17.5 (-57.2-12.2) | -33.9 (-62.9 to -16.5) | -2.3 (-30.3-17.4) | 0.022 |
|  | Nitrites | 15.9 (-6.6-46)^a^ | 25.4 (-1-47.4) | 14.5 (-23.3-42.8)^a^ | 0.238 |
|  | Nitrites/nitrates | 19.7 (-7.8-38.7) | 25.9 (-14-39.5) | 17.7 (-6.5-46.8) | 0.891 |
| Hypoxia | Catalase activity | 34.4 (-1.8-72.1) | 42.9 (10.4-78) | 26.6 (-6-70.7) | 0.453 |
|  | GPX1 activity | -26.1 (-33.8 to -12.5) | -30.4 (-41.1 to -12.5) | -23 (-32.9 to -12.1) | 0.304 |
|  | SOD2 activity | 8 (-17.6-24.3) | -11.5 (-26.8-32.9) | 9.3 (-9.5-22.7) | 0.171 |
|  | FRAP | -6.7 (-20.7-6.3) | -3.4 (-20.7-4.9) | -7 (-20.5-8.7) | 0.963 |
|  | AOPP | 23.3 (8-37.2) | 17.7 (9.6-31.9) | 24.4 (5.2-53.4) | 0.491 |
|  | MDA | 6.4 (-11.6-33.6) | 10.6 (-13.7-42.5) | 0.7 (-10.5-30.4) | 0.680 |
|  | Nitrotyrosine | -16.7 (-72.9-24.9)^a^ | -57.3 (-87.5-15.5) | 1.2 (-55.2-28.6)^a^ | 0.096 |
|  | Nitrites | -3.2 (-13.8-24.7)^a^ | -1.9 (-21.6-27.5) | -3.5 (-12.8-22.5)^a^ | 0.849 |
|  | Nitrites/nitrates | 11.8 (-5.1-43.9)^a^ | 10.9 (-12.3-41.7) | 12 (-4.7-61.4)^a^ | 0.751 |

^a^data missing for 1 participant

AOPP: advanced oxidation protein products, FRAP: ferric reducing antioxidant power, GPX1: glutathione peroxidase, MDA: malondialdehyde, SOD2: superoxide dismutase

Supplementary Table S2: Association of selected SNPs in antioxidant genes with relative change (%) of oxidative stress parameters after graded exercise test (all participants, N=37).

| Condition | Parameter | *CAT* rs1001179 | | | *GPX1* rs1050450 | | | *SOD2* rs4880 | | |
| --- | --- | --- | --- | --- | --- | --- | --- | --- | --- | --- |
|  |  | CC | CT+TT | P | CC | CT+TT | P | CC | CT+TT | P |
| Normoxia | Enzyme activity (CAT, GPX1 or SOD2)* | 49.9 (0.3-126.5) | 68.9 (17.8-113.3) | 0.435 | -17.4 (-38.5 to -4.6) | -12.8 (-29.2 to -6.4) | 0.424 | 12.3 (-6.6-36.8) | 12.5 (-8.8-68.9) | 0.986 |
|  | FRAP | -7.6 (-13.9-1.2) | -9.6 (-34.1 to -4.1) | 0.237 | -8.4 (-15.8 to -2.8) | -9.6 (-13.2-0.4) | 0.707 | -11.2 (-23.8-90) | -8.7 (-14.2 to -3.5) | 0.768 |
|  | AOPP | 27 (-0.1-96.1) | 58.1 (5.7-73.6) | 0.772 | 27 (1.3-84.2) | 45.7 (5.9-84.4) | 0.478 | 45.7 (5.9-91.9) | 30.7 (1.3-75.6) | 0.589 |
|  | MDA | 11.7 (-10.1-31.5) | -0.8 (-44.4-43.7) | 0.334 | 0.2 (-31.3-22.1) | -0.4 (-8.2-57.8) | 0.177 | -1.1 (-16.6-24.1) | 6.3 (-18.9-41.1) | 0.475 |
|  | Nitrotyrosine | -18.5 (-56.7-13.8) | -17.5 (-62.9-12.1) | 0.867 | -23.6 (-55.7-6.6) | -17.2 (-57.6-12.3) | 0.707 | -30 (-45.1-17.6) | -16.8 (-61.7-11.1) | 0.986 |
|  | Nitrites | 14.5 (-12.9-54.1) | 16.1 (0.7-47.4) | 0.825 | 14.2 (-16-46) | 16.9 (-2.8-49.1) | 0.694 | 25.4 (10.1-52.9) | 14 (-18.9-47.4) | 0.205 |
|  | Nitrites/nitrates | 14.2 (-8.9-28) | 26.5 (-8.4-73.8) | 0.319 | 24.6 (-4-39.1) | -2.7 (-16.6-37.6) | 0.097 | -4.6 (-24.5-66.1) | 20.1 (-5.7-39.1) | 0.392 |
| Hypoxia | Enzyme activity (CAT, GPX1 or SOD2)* | 30.0 (-6.0-58.8) | 48.0 (7.9-113.2) | 0.290 | -16.3 (-31.2 to -10.0) | -29.4 (-34.9 to -17.0) | 0.209 | -7.5 (-23.9-26.4) | 8.4 (-16.3-26.0) | 0.433 |
|  | FRAP | -6 (-20.8-8) | -7.3 (-20.7-5) | 0.891 | -0.7 (-11.8-11.2) | -19.1 (-31.7 to -2.3) | 0.017 | -20.7 (-34.9-8.4) | -6 (-16-7) | 0.392 |
|  | AOPP | 18.9 (9.3-32.6) | 25.4 (5.2-49.3) | 0.400 | 21.9 (6-28.5) | 23.3 (10.5-52) | 0.373 | 29.3 (0.4-53.9) | 22.2 (7.8-37.7) | 0.614 |
|  | MDA | 4.1 (-10.6-26.1) | 25.4 (-13.7-37.7) | 0.366 | 0.3 (-12.6-23.1) | 25.4 (-11.3-44.6) | 0.283 | 29.9 (-11.3-61.7) | 2.2 (-12.6-30.4) | 0.319 |
|  | Nitrotyrosine | 2.5 (-60.6-24) | -54.9 (-80.3-26.2) | 0.253 | -8.9 (-73.6-25.9) | -24.5 (-66.6-24.1) | 0.802 | 15.5 (-55-30.2) | -27.2 (-74.1-25.9) | 0.295 |
|  | Nitrites | 7.5 (-8.2-29.3) | -8.2 (-38.6 to -2.4) | 0.017 | -6 (-21.6-7.3) | 16 (-12.8-40.5) | 0.093 | 0.9 (-17-27.6) | -3.5 (-14.9-21.3) | 0.802 |
|  | Nitrites/nitrates | 11.3 (-7.9-42.9) | 18.3 (4.4-53.4) | 0.470 | 10.9 (-5.6-32.6) | 20.5 (-8-77.1) | 0.552 | 12 (3.8-59.2) | 11.6 (-6.4-41.7) | 0.774 |

*the activity of the enzyme, encoded by the corresponding gene: e.g. catalase activity for *CAT* rs1001179, glutathione peroxidase activity for *GPX1* rs1050450 superoxide dismutase activity for *SOD2* rs4880

AOPP: advanced oxidation protein products, FRAP: ferric reducing antioxidant power, MDA: malondialdehyde, SNP: single nucleotide polymorphism
